# Ultrasonographic Findings of Exocrine Pancreatic Insufficiency in Dogs

**DOI:** 10.3390/vetsci9080407

**Published:** 2022-08-04

**Authors:** Tina Pelligra, Caterina Puccinelli, Veronica Marchetti, Simonetta Citi

**Affiliations:** Affiliation Veterinary Teaching Hospital Mario Modenato, Department of Veterinary Sciences, University of Pisa, Via Livornese Lato Monte, 56121 Pisa, Italy

**Keywords:** exocrine pancreatic insufficiency, abdominal ultrasound, pancreatic acinar atrophy, chronic pancreatitis, dog

## Abstract

**Simple Summary:**

Exocrine pancreatic insufficiency (EPI) is a syndrome characterized by insufficient synthesis of pancreatic enzymes leading to clinical symptoms of malabsorption; this pathology has also been described in dogs. No studies are present about ultrasonographic appearance of the pancreas in the course of EPI in dogs. The purpose of this retrospective study was to describe ultrasound features of the pancreas during EPI in this species. In our study, the pancreas had a significantly lower thickness compared to the reference values of healthy dogs previously proposed; however, most of the dogs did not have other ultrasonographic pancreatic alterations. Moreover, in 85% of dogs, ultrasonographic intestinal abnormal findings were also identified. Based on our study, ultrasound could be a useful tool in the diagnosis of EPI. A normal but thinned pancreas associated with sonographic intestinal signs of inflammatory bowel disease in dogs with supportive clinical signs should suggests a diagnosis of EPI.

**Abstract:**

Exocrine pancreatic insufficiency (EPI) is a syndrome characterized by insufficient synthesis of pancreatic enzymes leading to clinical symptoms of malabsorption and maldigestion. There are no studies about ultrasonographic appearance of the pancreas with EPI in dogs. The purpose of this retrospective study was to describe ultrasound features of the pancreas during EPI in this species. Dogs with history and clinical signs of maldigestion, serum canine trypsin-like immunoreactivity (cTLI) values <5 µg/L, and abdominal ultrasound exam were included in the study. Size, shape, margin, echogenicity, echostructure, and pancreatic duct appearance of the right pancreatic lobe were valued. Additional sonographic intestinal findings were recorded. Thirty-four dogs were included. The mean pancreatic thickness in our population was significantly lower than the mean reference values of healthy dogs. In 68% of dogs, the pancreas had a normal ultrasound appearance. Ultrasonographic intestinal abnormal findings were identified in 85% of dogs and were suggestive of inflammatory bowel disease. Despite the fact that EPI is a functional diagnosis, ultrasound evaluation should be considered among the useful tests. The finding of a normal but thinned pancreas associated with sonographic intestinal signs of inflammatory bowel disease in dogs with typical history and supportive clinical signs could suggest a diagnosis of EPI.

## 1. Introduction

Exocrine pancreatic insufficiency (EPI) in dogs is a syndrome characterized by insufficient synthesis of digestive enzymes and secondary typical clinical signs of maldigestion [1].

This syndrome can be caused by two different diseases, one inherited and one acquired. The first is an autoimmune disorder, called pancreatic acinar atrophy (PAA), and it is caused by lymphocytic infiltration of the pancreatic acinar tissue, which leads to active tissue destruction [2,3]; the second is rarer and can be caused by chronic pancreatitis (CP) or pancreatic neoplasia [4]. The most common cause of EPI in dogs is PAA [1], while CP is reported to be an uncommon cause [5], in contrast to humans and cats [6,7,8].

The most common clinical signs of EPI, present in more than 90% of affected dogs, have been reported to be polyphagia, increased fecal volume and defecation frequency, weight loss, flatulence, poor digestion, loose, pulpy feces, and diarrhea. Other reported clinical signs are skin disorders, such as poor coat and eczema, and nervousness or aggressiveness that was also reported in one third of dogs with EPI [1,2,3,4,5,6,7,8,9].

The conclusive diagnosis for EPI is performed with measurement of serum canine trypsin-like immunoreactivity (cTLI). The reference range for cTLI in healthy dogs is between 5.0 and 35 µg/L [10]. Very low serum cTLI concentrations (<2.5 µg/L) associated to typical clinical signs are considered highly diagnostic for severe EPI, with a sensitivity of 100% [10]. However, low serum cTLI values (<5.0 µg/L) repeated over time in some clinically healthy dogs can be indicative of subclinical EPI, and in these cases monitoring is needed [11]. Furthermore, the use of serum cTLI to diagnose EPI in dogs with CP can be difficult, and its sensitivity is <100% because it could produce a normal result in case of concurrent pancreatic inflammation, which increases its value [12].

Generally, the lower the cTLI value, the more accurate a single measurement is in assessing pancreatic dysfunction; however, in cases of subclinical EPI, limitations could be present in the use of serum cTLI. Overlapping cTLI results between subclinical and normal dogs are also to be expected, so a normal cTLI value (>5.0 lg/L) does not rule out the presence of mild to moderate pancreatic dysfunction [1]. In this cases, ultrasonographic abdominal evaluation could be useful. 

Regarding the appearance of the pancreas in course of EPI, the macroscopic and histological findings are widely described in the literature. The progression of the PAA was divided into a subclinical phase (characterized by partial acinar atrophy, lymphocytic infiltration, and absence of fibrosis) and clinical phase (characterized by severe end-stage atrophy) [4]. 

In the course of the subclinical phase, both atrophied and normal acinar parenchyma were found, and the pancreatic volume is reduced with the presence of scattered areas of atrophied tissue among the normal tissue. The histologic findings in this phase were typical for an autoimmune disease [4,13,14]. 

In dogs with end-stage PAA, at the pathologic examination, the pancreas appears very thin, and the normal glandular structure is hardly recognizable, the length is normal, and the pancreatic duct is prominent [14]. Histologically the normal acinar parenchyma is absent and replaced by adipose tissue, while an increase of fibrous tissue is generally not observed, and the endocrine tissue is not involved [1]. 

The pathologic findings in CP are different from those of PAA. The pancreas can appear macroscopically normal; however, when it loses 80 to 90% of lipase production and islet cell function, EPI can develop and the pancreas can become obviously nodular with a variety of combinations of massive scarring, intensified lobular appearance, interstitial fibrosis, loss of parenchyma, and adhesions to the surrounding organs [1,15,16]. The histopathological findings of end-stage CP can be an extensive destruction of both endocrine and exocrine tissue, inflammatory cells in the interstitium, and marked interlobular and intralobular fibrosis [4,5].

Ultrasound imaging of the pancreas is an important non-invasive technique in the diagnosis of pancreatic disease. One study reported that the thickness of the canine pancreas ranged from 3.5 to 16 mm, with a reference value of approximately 1 cm in medium-sized dogs (15–30 kg) [17]. The mean diameter of the pancreatic duct is reported to be 0.8 mm in medium-sized dogs, with a range of 0.1 to 1.2 mm in all dogs [17]. The role of ultrasound in the diagnosis of CP has been poorly investigated [18]. Watson et al. 2010 defined that ultrasound sensitivity for the diagnosis of CP in dogs is 56% (35–75%) due to the few ultrasonographic changes secondary to the low-grade inflammation, tissue loss, and fibrosis. The ultrasonographic findings of CP previously described were decreased pancreatic size, variable mixed echogenicity of pancreatic parenchyma, diffuse hyperechogenicity, nodular echotexture, hyperechoic foci (possibly due to mineralization, fat, or fibrosis), scarring, and irregular widening of the pancreatic ducts [18,19]. In end-stage CP, the atrophic pancreatic tissue may be not visible ultrasonographically and the use of endoscopic ultrasound could increase the sensitivity of diagnosis [20]. Two studies described the endoscopic ultrasonographic changes of pancreas in dogs after experimental pancreatic duct legation and obliteration to obtain CP, with post-mortem histologic diagnosis [21,22]. The images showed a dilated pancreatic duct, increased parenchymal echogenicity, lobularity, increased echogenic septations, visible pancreatic duct side branches, irregular margins of the main pancreatic duct, hypoechoic foci, and echogenic foci [21,22].

To our knowledge, there are no specific reports on ultrasonographic findings in dogs with EPI. Only in one study of about 14 cases of CP in dogs, of which 5 had EPI, ultrasound examination of pancreas was reported: in four dogs, the pancreas appeared unremarkable on the ultrasound, while in one dog the pancreas appeared abnormal [15].

Our hypothesis was that, in most cases, the ultrasound appearance of the pancreas with EPI could be normal but that in many cases this may appear thinned. We also hypothesize that ultrasound signs of inflammatory bowel disease are present in many cases of EPI. The proposal of our study is to suggest the suspicion of EPI in cases where abdominal ultrasound detects the thinned pancreas associated with signs of intestinal inflammation. 

## 2. Materials and Methods

This retrospective, descriptive study was performed at the Veterinary Teaching Hospital of the Department of Veterinary Sciences, University of Pisa. 

The electronic medical record database was searched over an 11-year period (2010–2021) for patients with EPI and abdominal ultrasound performed at the time of the diagnosis. The diagnosis of EPI had been made based on history, clinical signs, and serum cTLI concentration <5.0 µg/L. The abdominal ultrasound examination had to consist of a complete report with the description of the pancreas and images or videos of the right lobe of the pancreas in order to evaluate its appearance.

Due to the retrospective study design, no institutional animal care approval was required. However, all owners signed a consent form for use of image and medical information. The use of data was approved by the hospital.

The following information were recorded for each patient: age, breed, sex, body weight (BW), and body condition score (BCS). In our study, the dogs were classified, according to their BW, into 2 groups: less than 15 kg (Group 1) and more than or equal to (≥) 15 kg (Group 2).

The evaluation of BCS was performed using a validated BCS system, which is a nine-point rating scale with a range of categories from cachectic to severely obese [23]. 

Transcutaneous abdominal ultrasound was performed by 2 experienced ultrasonographers (S.C and T.P) with a Canon Aplio a CUS-AA000 (Canon Medical Systems Europe B.V., Zoetermeer, the Netherlands), with a 7.5 MHz microconvex probe and a 12 MHz linear probe. DICOM images and video files were stored in our hospital database and retrospectively reviewed by one author (T.P). A single individual (T.P.) was used to retrospectively assess the images and videos to prevent interobserver variation from impacting the data.

Size, shape, margin appearance, echogenicity, echostructure, and presence of parenchymal alterations (nodules or cists) were recorded. Pancreatic duct, when visible, was valuated in course, intraluminal content, and walls.

**Figure 1:**
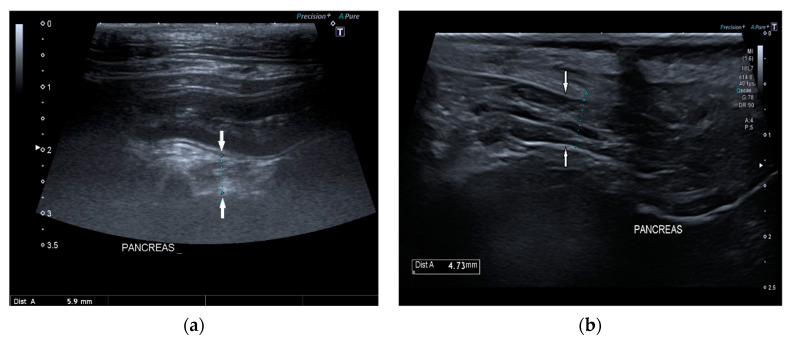
Ultrasound images (**a**) and (**b**) of the right pancreatic limb (between white arrows) with caliper measurement.

Pancreatic size was determined using calipers on a video frame or a still image. As described in the previous study, given that the pancreas has a triangular shape, it was considered a good compromise to measure its thickness perpendicular to the long axis [17]. Due to the low identification rate of the left pancreas in dogs, the pancreatic thickness was evaluated by measuring the right lobe located ventromedial to the right kidney and medial to the descending duodenum (Figure 1) [17]. 

The shape was subjectively assessed as normal or altered, and the margins as regular and smooth or irregular. Pancreatic echogenicity was compared to the liver and were classified as isoechogenic or diffuse hyperechogenic. Pancreatic echostructure was classified as homogeneous, heterogeneous, nodular, and/or with acoustic shadowing due to mineralization and scarring. Pancreatic duct was measured, when possible, and classified as following: linear or tortuous course, anechoic content or intraluminal debris, and thin or thick walls.

Alterations related to the gastrointestinal tract were also reviewed. In the intestinal tract, peristalsis (normal or reduced), intestinal mucosal pattern (normal, hyperechoic, speckled, or striated), intestinal wall thickness (normal or increased), were considered. Presence of peritoneal effusion and lymphadenopathy were detected if present.

All statistical analysis were carried out by one of the authors (C.P.), using commercially available statistics software (GraphPad, GraphPad Software Inc, San Diego, CA, USA). 

The Shapiro–Wilk test was used to assess the distribution of data for age, BW, BCS, pancreatic size and TLI values. Means ± SD and medians with ranges were calculated depending on data distribution. 

The Spearman test was performed to evaluate a possible correlation between right pancreatic lobe thickness and BW, BCS, and age. 

The Mann–Whitney test was used to evaluate the possible difference between the BW of dogs in which the pancreas was visualized and the BW of those in which it was not identified. Furthermore, this test was used also to evaluate if there was a significant difference between the median values of pancreatic right lobe thickness in the two weight groups.

The possible association between pancreas visualization, age, and BW was assessed using Fisher’s exact test.

The Student’s t-test (using MedCalc Software Ltd. Comparison of means calculator. https://www.medcalc.org/calc/comparison_of_means.php Version 20.027; accessed on 25 February 2022) was used to test whether the mean right pancreatic lobe thickness and mean pancreatic duct diameter in our population with EPI differed from the mean reference values proposed by Penninck et al. [17]. 

A *p*-value of <0.05 was considered statistically significant. 

## 3. Results

Thirty-four dogs were included. The breeds included were German Shepherd (*n* = 12), Epagneul Breton (*n* = 3), Rottweiler (*n* = 2), Shih-Tzu (*n* = 2), Collie (*n* = 2), mix breed dogs (*n* = 7), and one of each breed of the following: Boxer, Jack Russel Terrier, Czechoslovakian Wolfdog, Labrador Retriever, French Bulldog, and Cao de Agua Portugues.

Sixteen dogs (47%) were male and 18 (53%) were female. Of the female dogs, 67% (12/18) were intact and 33% (6/18) were spayed. All male dogs were intact. Ages ranged between 3 months and 13 years, with a mean of 5.9 +/− 3.5 years.

The mean age of the dogs at the onset of signs was 5.5 years, and the age distribution is reported specifically in Table 1.

The weight of the population ranged between 3.1 kg and 49.2 kg, with a mean of 20.6 kg. Thirty-two percent of the population had a weight less than 15 kg (11/34 dogs) and 68% (23/34) of the population was more than 15 kg. The median BCS of the population was 3/9 (range: 2/9–5/9). 

The clinical signs observed in dogs of our study are reported in Table 2. 

The median serum TLI of the population was 1.4 µg/L (range: 1–5 µg/L). In 24 cases (70.6%), the TLI was less than 2.5 µg/L, while in the remaining 10 cases (29.4%) it was between 2.5 µg/L and 5 µg/L. 

In the reviewed images, the right lobe of the pancreas was identified in 64.7% (22/34) of dogs. The reports showed that the pancreas could not be visualized in 12/34 dogs (35.3%). In 18/22 dogs, it was possible to measure it. The comparisons between the right pancreatic lobe thickness, the pancreatic duct diameter in our population with EPI, and the mean reference values proposed by Penninck et al., 2013 [17] are summarized in Table 3. 

Pancreas thickness in subjects with TLI less than 2.5 µg/L was 5.1 mm and in dogs with TLI between 2.5 µg/L and 5 µg/L was 6.2 mm.

A statistically significant difference was found between the BW of dogs in which the pancreas was visualized and the BW of those in which it was not identified (*p* = 0.0032). The pancreas in dogs with a weight <15 kg has always been visualized, while it was not seen in 43.5% of dogs with a weight ≥15 kg. Dogs in which the pancreas was not visualized had a median BW of 25.5 kg (range: 19–49.2 kg), while dogs in which the pancreas was identified had a median BW of 14.8 kg (range: 3.1–40.6 kg).

No significant difference was found between the median values of pancreatic right lobe thickness between the two weight groups (*p* = 0.5857). No significant correlations were found between pancreatic thickness and BW (*p* = 0.87), BCS (*p* = 0.3574) or age (*p* = 0.0756). No association was found between pancreas visualization with age (*p* = 0.7041). 

Of the 22 identified right pancreatic lobes, 13 (59%) had images of the pancreatic duct available for review. The mean pancreatic duct diameter was 0.65 ± 0.17 mm and the reported mean measurement in healthy dogs was 0.7 ± 0.2 mm. No significant difference was found between these values (*p* = 0.3789).

The course of the pancreatic duct was linear in 77% (10/13) and tortuous in 23% (3/13); its content was anechoic in all cases (13/13); the walls were thin in 92% (12/13) and dilatated in 8% (1/13). 

**Figure 2:**
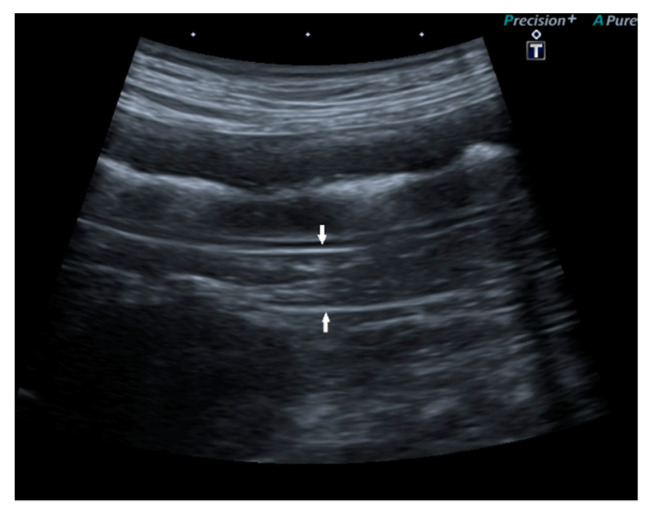
Ultrasound image of the right pancreatic limb with normal shape and echogenicity, regular margin, and homogeneous echotexture.

In 15/22 dogs (68%), the pancreas had a normal ultrasound appearance, with normal shape, margins, and echogenicity, homogeneous echotexture, and without pancreatic duct abnormalities (Figure 2). 

**Figure 3:**
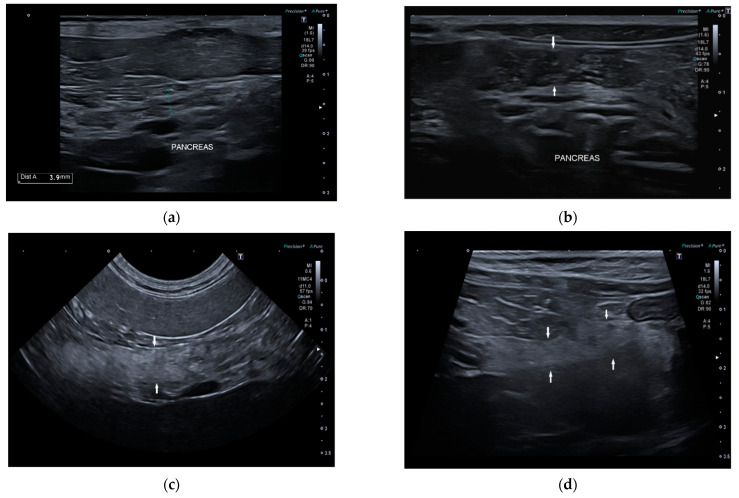
Ultrasound image of right pancreatic limb: (**a**) Pancreas (between the caliper) with regular margin, homogeneous echogenicity, and mild inhomogeneous echotexture (**b**) Pancreas (between the white arrows) with regular margins (thickness 6.5 mm), normal echogenicity, and multiple point mineralizations (**c**) Pancreas (between the white arrows) with irregular margins (thickness 5 mm) multifocal hyperechogenicity, and inhomogeneous echotexture (**d**) Pancreas (between the white arrows) with irregular margin (thickness 4 mm), diffuse and severe parenchymal hyperechogenicity, and inhomogeneous echotexture.

Conversely, 7/22 dogs (32%) had one or more of the anomalies described: two dogs had multiple hyperechoic spots in pancreatic parenchyma; one dog had inhomogeneous pancreatic echotexture with multiple hyperechoic spots; one dog had diffuse hyperechoic parenchyma with inhomogeneous echotexture and tortuous pancreatic duct; one dog had irregular margins and shape, hyperechoic parenchyma, and inhomogeneous echotexture; one dog had diffuse pancreatic heterogenicity, and one dog had irregular margins and tortuous pancreatic duct (Figure 3). The age of onset of symptoms in six of these seven dogs with abnormal parenchyma was greater than 4 years.

**Figure 4:**
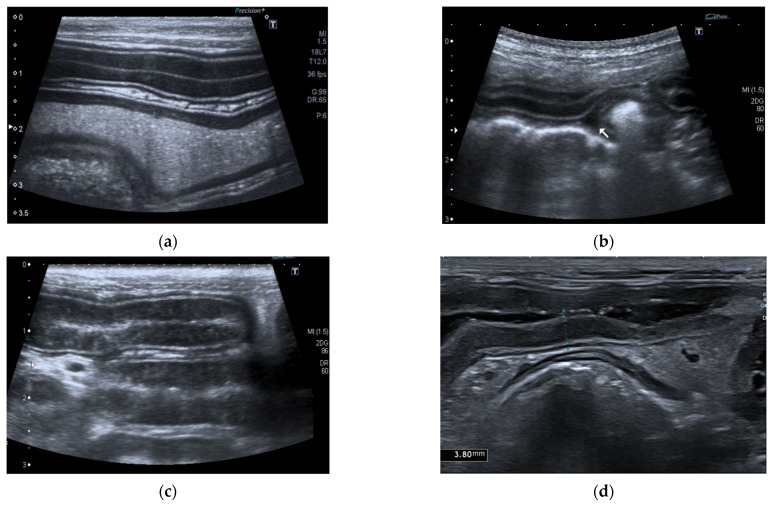
Ultrasonographic intestinal abnormal findings. (**a**) Jejunal dilatation with fluid content; (**b**) Mild abdominal effusion; (**c**) Jejunal segment with hyperechoic striation within the mucosa; (**d**) Jejunal segment with hypoechoic/nearly anechoic mucosa and fluid content.

Ultrasonographic intestinal abnormal findings were identified in 85% (29/34) of dogs, and the ultrasonographic findings are summarized in Table 4 (Figure 4).

## 4. Discussion

Canine exocrine pancreatic insufficiency (EPI) is a disease characterized by insufficient production of digestive enzymes by pancreatic acinar cells. EPI is a functional diagnosis based on typical findings in clinical history and clinical signs and measuring decreased pancreatic secretion capacity by pancreatic function tests. To date, cTLI measurement is the most useful functional test for diagnosing abnormal pancreatic function in dogs [1]. 

In our study, we decided to consider the cases of EPI with values of cTLI <5, in order to include both the cases in which the diagnosis was certain with cTLI <2.5 and the cases in which the diagnosis was doubtful with values between 2.5 µg/L and 5 µg/L. This choice is due to the need to also include early-stage cases of EPI and cases of subclinical EPI for partial pancreatic atrophy or chronic pancreatitis.

From an epidemiological point of view, our data confirm what is reported in the literature: German Shepherds were the most affected breed (35%), but no other breed predisposition was identified in this study. Associations with EPI and dog breed were found in German Shepherds, rough-coated collies, Chow Chows, and Cavalier King Charles Spaniels [4,5,6,7,8,9]. PAA is the most common cause of severe EPI in German Shepherds [4,9,24], rough-coated collie [25,26], and English setter [27]. There are strong breed associations with CP in the English cocker spaniel, Cavalier King Charles Spaniel, Boxer, collie, and Jack Russell Terrier [9,10,11,12].

The type and frequency of clinical signs reported in our study are comparable to those described in the literature [1]. In our study, although there are some clinical signs more frequently present in cases of EPI, it is established that they are not pathognomonic for exocrine pancreatic dysfunction, as some diseases of the small intestine could cause similar signs of malabsorption or indigestion [1]. In some cases, the symptoms manifested severely during the first years of life, while in other cases the dogs had no symptoms for many years and clients were unable to specify when the onset of the disease has occurred. In these subclinical cases, despite low cTLI values and markedly macroscopic changes in the pancreas, there are no suggestive clinical signs or chronic and intermittent gastrointestinal signs not typical of EPI. In dogs with supporting clinical signs of EPI, it is not possible to distinguish between PAA and CP, because in the final stage of both diseases, the symptoms are the same. In cases of PC, the clinical history may be useful for diagnostic suspicion because intermittent and low-grade gastrointestinal symptoms are commonly present, although some may present acutely with acute pancreatitis, extrahepatic biliary obstruction, or even acute diabetic ketoacidosis crisis. [12]. About BCS, in our study most of the patients had excessive thinness (median BCS 3/9). As reported by previous studies, severe weight loss is one of the main symptoms of this disease [1]. 

In our study, the age of onset of EPI does not allow to distinguish between PAA and PC, as described by Watson 2003. The clinical signs of EPI caused by PAA are usually seen in young adults 1 to 4 years of age (93% of cases), although sometimes, in the case of subclinical EPI caused by partial PAA, the clinical disease may develop later in life [1,4,28]. The rate of progression of this pathology, however, is not predictable, so the same cases of partial PAA may not show symptoms for many years or have sporadic or chronic gastrointestinal signs or have cTLI concentrations in range of 2.5 to 5.0 µg/L [4]. The onset of EPI in the case of CP is variable due to the rapidity of pancreatic destruction and depends on the frequency and severity of attacks of pancreatitis [5]. CP has already been described in the past by Ettinger and Anderson in 1972 as a possible cause of EPI, and other authors also described it as a possible cause even if underestimated [29,30,31]. In these cases, the pancreas could be greatly diminished in size, even virtually absent, but because the intensity of inflammation was not great at any given episode, the clinical signs of pancreatitis were never severe, and the dog is already presented with typical clinical signs of EPI [16]. 

Both in cases of subclinical PAA and in cases of CP, only through repeated measurements of the cTLI value it is possible to monitor the progress of pancreatic function and identify subclinical cases [4]. In our study, in 55% of cases the age on onset of EPI was more than 4 years old. Therefore, this suggests that more than half of EPI cases are underestimated in the subclinical phases, both in the case of PAA and in the case of CP. This finding suggests that regardless of the patient’s age, the diagnosis of EPI must always be considered among the differential diagnoses in case of signs referable to maldigestion.

Ultrasonographic exam of the pancreas plays a controversial role.

It is known that the ultrasound examination during acute pancreatitis has a sensitivity of 68% [32], while during chronic pancreatitis the sensitivity is 56% [12]. Furthermore, there are no data in literature regarding the ultrasound examination of the pancreas in EPI. According to our study, the ultrasound evaluation of the pancreas during EPI is not a reliable diagnostic technique since the diagnosis of this disease is functional. The usefulness of this technique could be to suggest the presence of a reduction of the pancreatic parenchyma in cases of subclinical EPI, partial pancreatic atrophy, or chronic pancreatitis [33].

The first difficulty is evidenced by the fact that in our patients the pancreas was visualized only in 64.7% of cases, despite the high-performance ultrasound equipment and the experience of the sonographers. A possible cause could be in some cases due to the severe intestinal meteorism and in other cases due to the extremely reduced thickness of the pancreas. However, most interestingly, the pancreas was seen in all subjects weighing <15 kg, while only in 43.5% of patients weighing ≥15 kg.

Pancreas thickness is also not correlated with BCS and BW in our dogs; this finding is opposed to what was reported in the 2013 Penninck study, in which the thickness of the pancreas in healthy dogs was positively correlated with BW in all lobes. This finding could indicate that, probably, in subjects with EPI the size of the pancreas is inadequate with respect to BW.

In a previous study, no significant correlation was found between age and pancreatic thickness in healthy dogs, as in cats [17,18,19]. Likewise, a significant correlation has not been demonstrated in our population. 

Another interesting result was that in 68% of cases, the pancreas was only reduced in size, while all the other ultrasound parameters were normal. This result is unexpected because we expected some more signs referable to CP. This finding agrees with previous pathological studies in which the pancreas during EPI due to PAA was extremely thin, devoid of glandular parenchyma and fibrous tissue [4]. In our study, among dogs with a TLI value <2.5 µg/L, suspected of PAA, it was possible to measure the thickness of the pancreas only in 4/15 cases and their mean was 5.1 mm, probably because it was severely thin. 

In only three subjects, the pancreas was hyperechoic and inhomogeneous, and in three other cases it presented hyperechoic foci of mineralization. In these cases, the age of onset of symptoms was greater than 4 years. This late onset of clinical manifestations, although pancreatic parenchyma atrophy begins in the early stages of the disease, may be due to the large functional reserve capacity of the pancreas [34]. In our study the pancreatic duct of the right pancreatic lobe was visualized and measured in 59% of cases and only in three cases it was tortuous and in one case dilatated. This data, associated with the low percentage of parenchymal anomalies, can be explained by considering that in dogs the most frequent cause of EPI is PAA in which the pancreas mainly evolves into parenchymal atrophy without fibrosis or calcifications. Abnormalities of the pancreatic duct, such as dilatation, tortuosity, and the presence of intraductal or parenchymal calcification/plugs are an important evaluation index of CP and EPI in humans and cats [8,9,10,11,12,13,14,15,16,17,18,19,20,21,22,23,24,25,26,27,28,29,30,31,32,33,34]. Our hypothesis is that in our study there are more cases of PAA than CP. This could be suggested by the fact that among the cases where the pancreas was seen, most showed small size and no abnormal parenchymal signs. Moreover, in the literature, PAA is more frequent and the pancreas with PAA appears morphologically thin and without parenchymal fibrosis.

Previously described pancreatic ultrasonographic findings in dogs with EPI have not been described in case of PAA, while in case of CP the information available is limited and includes: markedly thin to nearly absent pancreatic parenchyma, variable mixed echogenicity of pancreatic parenchyma, nodular echotexture, acoustic shadowing due to mineralization and scarring, and irregular widening of the pancreatic ducts [19]. In human medicine, an ultrasound score has been developed for ultrasound staging of CP, which includes the evaluation of the pancreatic size (normal, mild, diffuse, or complete reduction), the echostructure (homogeneous, mild, moderate, or severe inhomogeneity), the degree of ectasia of the duct (normal, mild, moderate, or severe), and the presence of calcifications/parenchymal plugs (absent, focal, segmental, or diffused) [34]. A perfect agreement was found between the score and the severity of CP [34]. Furthermore, in human medicine it is possible, through ultrasound and MRI techniques, to evaluate the reduction of pancreatic function and there is a correlation between ultrasound characteristics, the severity of CP, and the presence of EPI [34]. In our study, due to the small presence of parenchymal alterations, it is not possible to define this correlation. 

In our study, considering the prevalence of the predisposed breed and most of the cases with very low cTLI value, there are probably predominantly dogs with EPI due to PAA while, as described in the literature, it is rarer that there are cases secondary to CP. This could be the reason why no severe and widespread ultrasound changes of the pancreatic parenchyma were detected. For this reason, it is probable that the role of PAA is underestimated in clinic practice, considering that this diagnosis is closely correlated with the young age of onset.

In our study, the concurrent ultrsonographic signs mainly included gastrointestinal problems (85%) and the most common gastrointestinal findings were dilation of the intestinal loops, the presence of intestinal liquid content, reduced peristalsis, and hyperechogenicity of the intestinal mucosa. It is known that when pancreatic enzyme production is reduced, maldigestion occurs and it is associated with failure of intraluminal digestion due to abnormal activities and impaired function of mucosal enzymes, reduced degradation of exposed brush border proteins, reduced pancreatic trophic factors, and dysbiosis [35]. 

Despite this, these intestinal abnormal ultrasound findings are common to other small bowel diseases such as inflammatory bowel disease [36]; the high incidence of this finding in the case of EPI suggests that the ultrasound exam in the course of intestinal diseases cannot exclude the evaluation of the pancreas. If the pancreas were reduced in size, an evaluation of its function would be suggested.

Our study has some limitations. The first one is its retrospective design; moreover, the pancreatic duct was not viewable in every archived image, probably due to the presence of concomitant gastrointestinal abnormality or the large size of some patients.

Although EPI is a disease with a moderate incidence in dogs, the number of patients in our study is relatively low. This may be secondary to the fact that this condition is easily identified by primary care veterinarians and only the most complex cases are referred to our referral centre.

## 5. Conclusions

In conclusion, some interesting aspects emerged from this study, suggesting that the ultrasound evaluation should be considered among the tests for the diagnosis of EPI. Ultrasound examination cannot replace functional evaluation of the pancreas but can suggest an abnormal appearance or possible atrophy. In most of the patients whose pancreas was visualized, the pancreas was thinner than normal and free from parenchymal and duct abnormalities. From this reflection we support the hypothesis of the prevalence of PAA in dogs with EPI. Moreover, in 85% of cases intestinal ultrasound abnormalities were present. Therefore, a normal but thinned pancreas associated with sonographic intestinal signs of inflammatory bowel disease in dogs with supportive clinical signs should suggest a diagnosis of EPI.

## Figures and Tables

**Table 1:** Age of onset of signs of EPI in 34 dogs.

Age	N° of Dogs	% of Dogs
<1	5/34	15%
1–4	10/34	30%
>4	19/34	55%

**Table 2:** Clinical signs observed in 34 dogs.

Clinical Signs	N° of Dogs	% of Dogs
Diarrhea	27/34	79%
Weight loss	24/34	70%
Steatorrhea	18/34	53%
Flatulence	16/34	47%
Vomiting	14/34	41%
Skin disorder	10/34	29%
Polyphagia	7/34	20%
Polydipsia	6/34	17%
Coprophagia	6/34	17%
Dysorexia	2/34	6%

**Table 3:** Right lobe pancreatic thickness in 22 dogs with EPI compared to reported mean measurements in healthy dogs (20). In the reference study, the dogs of group 2 weighed between 15 and 30 kg, while in group 2 of our study, five dogs weighing more than 30 kg are included.

Dogs	Weight Category (kg)	Mean ± SD (mm)	Penninck et al., 2013 [17]	*p*
Group 1	<15	5.95 ± 1.5	7.25 ± 1.47	<0.0061
Group 2	>15	5.48 ± 1.3	8.62 ± 1.64	<0.0001
Total population		5.74 ± 1.4	8.1 ± 1.8	<0.0001

**Table 4:** Ultrasonographic gastrointestinal findings in 29 dogs with EPI.

Ultrasonographic Gastroenteric Findings	N° of Dogs	% of Dogs
Diffuse dilatation with fluid content	21	61%
Reduced peristalsis	21	61%
Hyperechoic mucosa	13	38%
Abdominal effusion	6	17%
Mucosal Lymphangiectasia	4	12%
Gastric or intestinal foreign body	3	9%
Jejunal lymphadenopathy	3	9%
Peritoneal reactivity	2	6%

## Data Availability

Not applicable.
